# Supplementary material for: Multi-targeted 1H/19F MRI unmasks specific danger patterns for emerging cardiovascular disorders
Source: Nat Commun. 2021 Oct 6;12:5847. doi: 10.1038/s41467-021-26146-6 (PMC8494909; doi:10.1038/s41467-021-26146-6)
Supplement: Supplementary file 1 — Supplementary Information [file 41467_2021_26146_MOESM1_ESM.pdf]

Supplemental Material

Multi-Targeted $^{1}H/^{19}F$ MRI Unmasks Specific Danger Patterns for Emerging Cardiovascular Disorders

Ulrich Flögel[1-3], Sebastian Temme[1,4], Christoph Jacoby[1,3], Thomas Oerther[5], Petra Keul[6], Vera Flocke[1], Xiaowei Wang[7], Florian Bönner[3], Fabian Nienhaus[3], Karlheinz Peter[7], Jürgen Schrader[2,3], Maria Grandoch[8], Malte Kelm[2,3], Bodo Levkau[6]

[1]Experimental Cardiovascular Imaging, Institute for Molecular Cardiology, Heinrich Heine University, Düsseldorf, Germany; [2]Cardiovascular Research Institute Düsseldorf (CARID), Heinrich Heine University, Düsseldorf, Germany, [3]Department of Cardiology, Pneumology and Angiology, University Hospital Düsseldorf, Germany; [4]Department of Anesthesiology, Heinrich Heine University, Düsseldorf, Germany; [5]Bruker BioSpin, Rheinstetten, Germany; [6]Institute of Molecular Medicine III, Heinrich Heine University, Düsseldorf, Germany; [7]Baker Heart and Diabetes Institute, Melbourne, Australia; [8]Department of Pharmacology and Clinical Pharmacology, Heinrich Heine University, Düsseldorf, Germany.

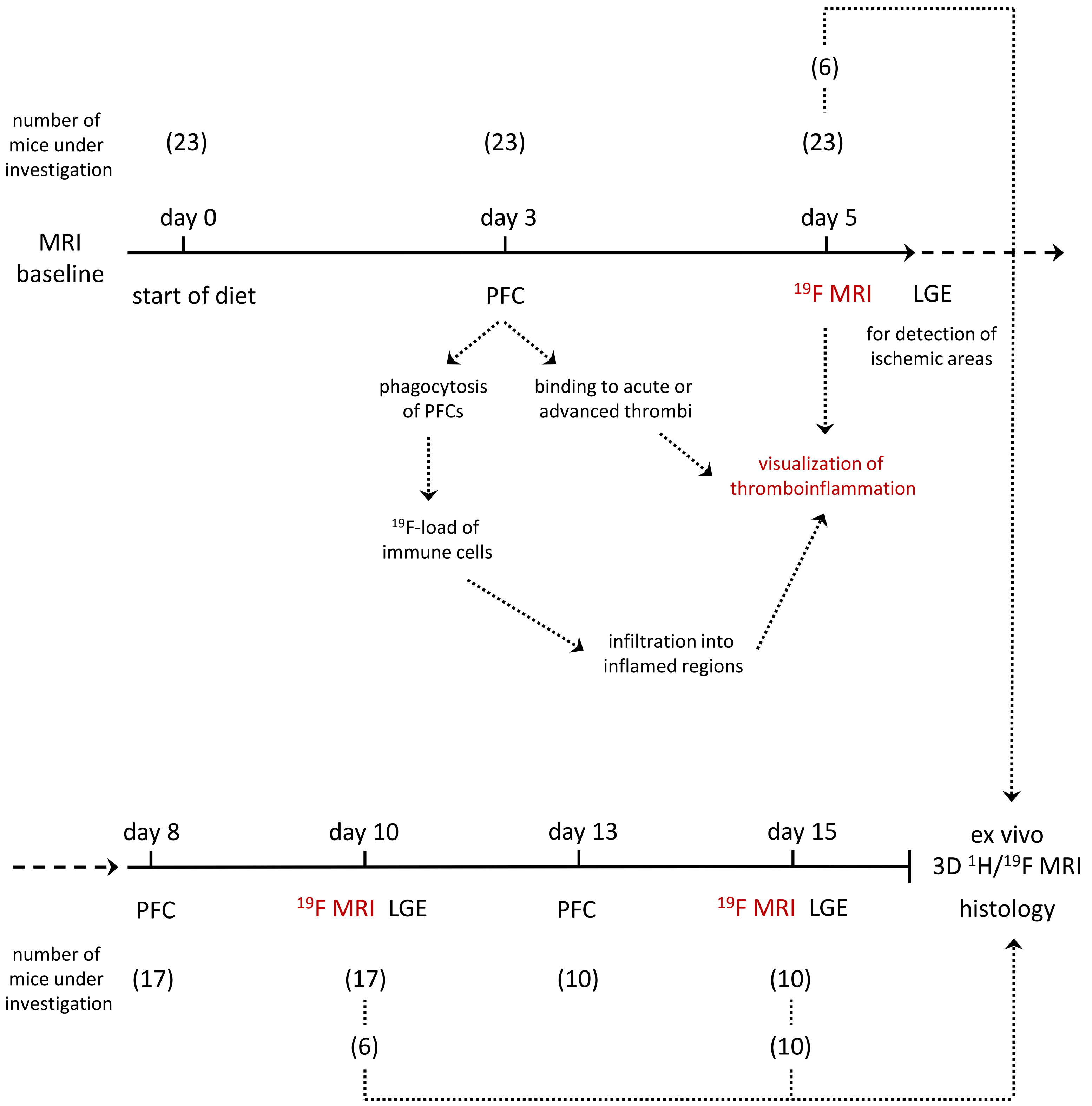

Supplementary figure 1: Experimental protocol of the main study

Animals were investigated at baseline and 5, 10 and 15 days after onset of the diet. Two days prior to MRI animals received intravenous PFC injections allowing for adequate loading of immune cells and/or binding to thrombi. Application of Gd-containing contrast agents for detection of ischemic areas by LGE was carried out after ^{19}F MRI to avoid alterations of ^{19}F relaxation times which may impact on quantification. Between day 10 and 13 one mouse died spontaneously.

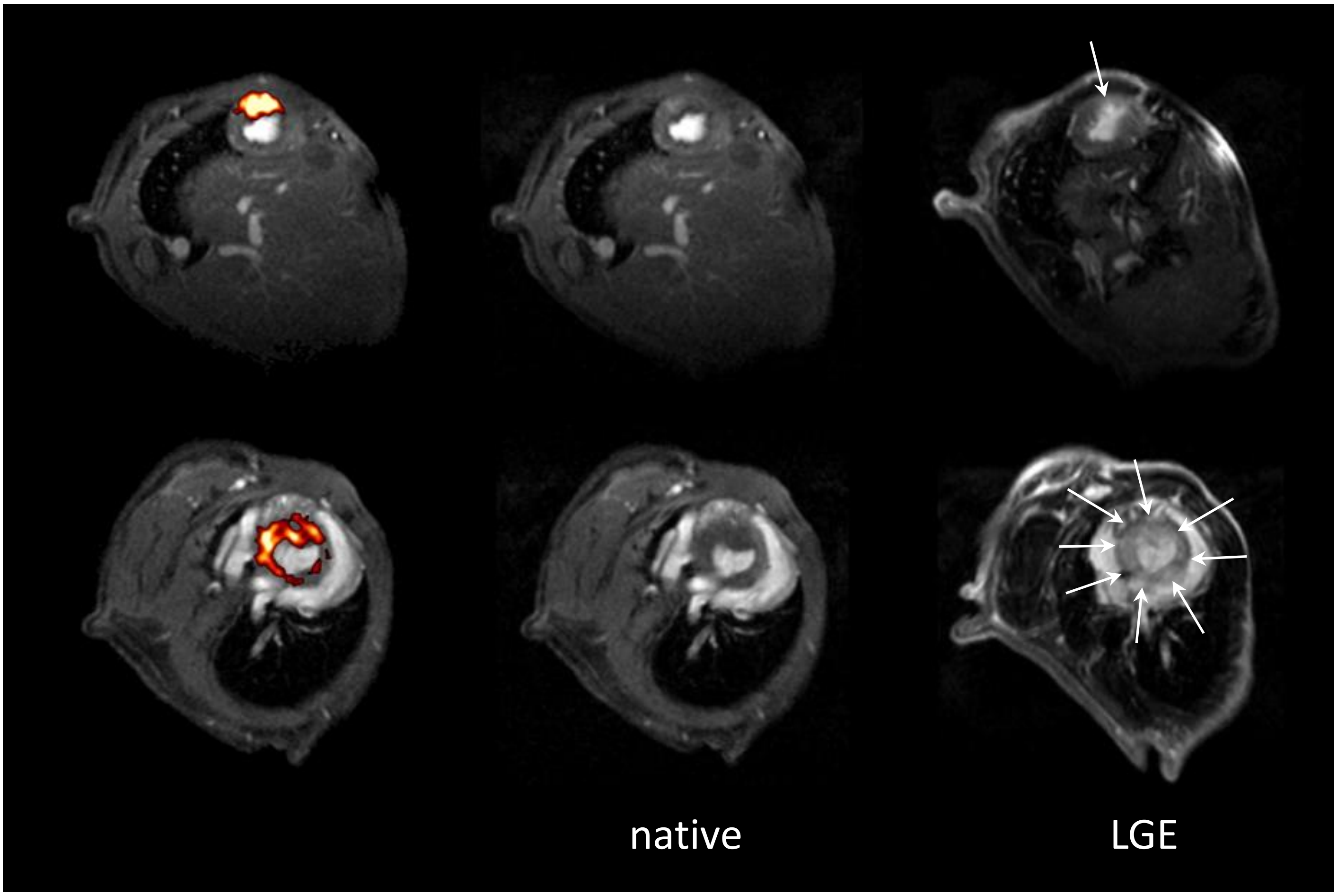

Supplementary figure 2: Further sites of inflammation and cardiac ischemia at day 15 after onset of the diet

Apical (top) and basal (bottom) short axis views demonstrate inflammation at base and apex of the heart with congruent LGE patterns (white arrows) at this late point in time. Application of Gd-containing contrast agents for detection of ischemic areas by LGE was carried out after ^{19}F MRI to avoid alterations of ^{19}F relaxation times which may impact on quantification of ^{19}F MR signals.

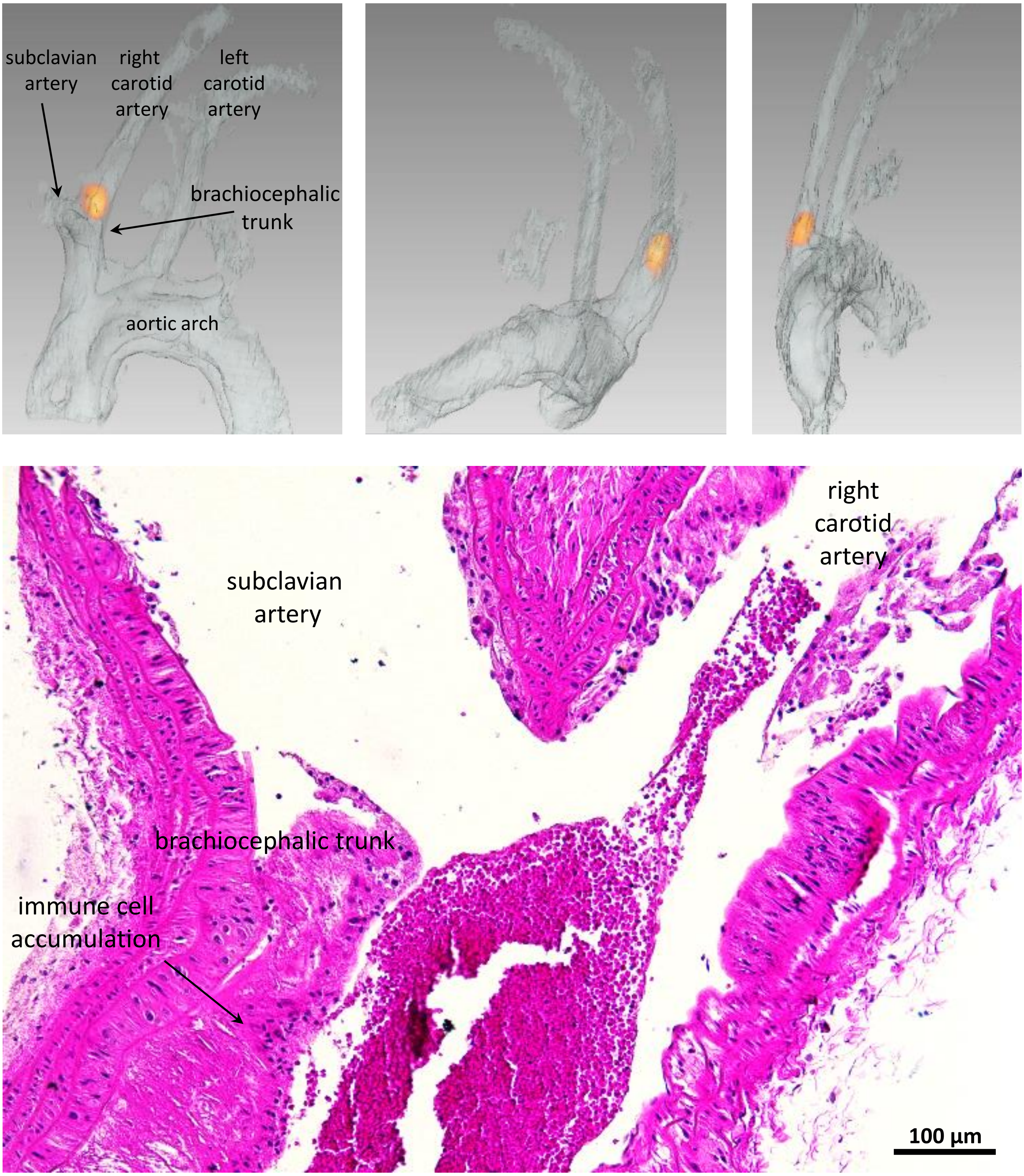

Supplementary figure 3: Validation of the ^{19}F signal and presence of immune cells within the aortic wall at day 5 after onset of the diet

Top: Different views from an ex vivo high resolution 3D ^{1}H/^{19}F MRI dataset confirming the location of the ^{19}F signal (red) at the luminal site of the brachiocephalic trunk. **Bottom:** Corroboration of infiltrated immune cells within the aortic wall at the lumnal site (arrow) near the brachiocephalic trunk by histology (10-fold magnification).

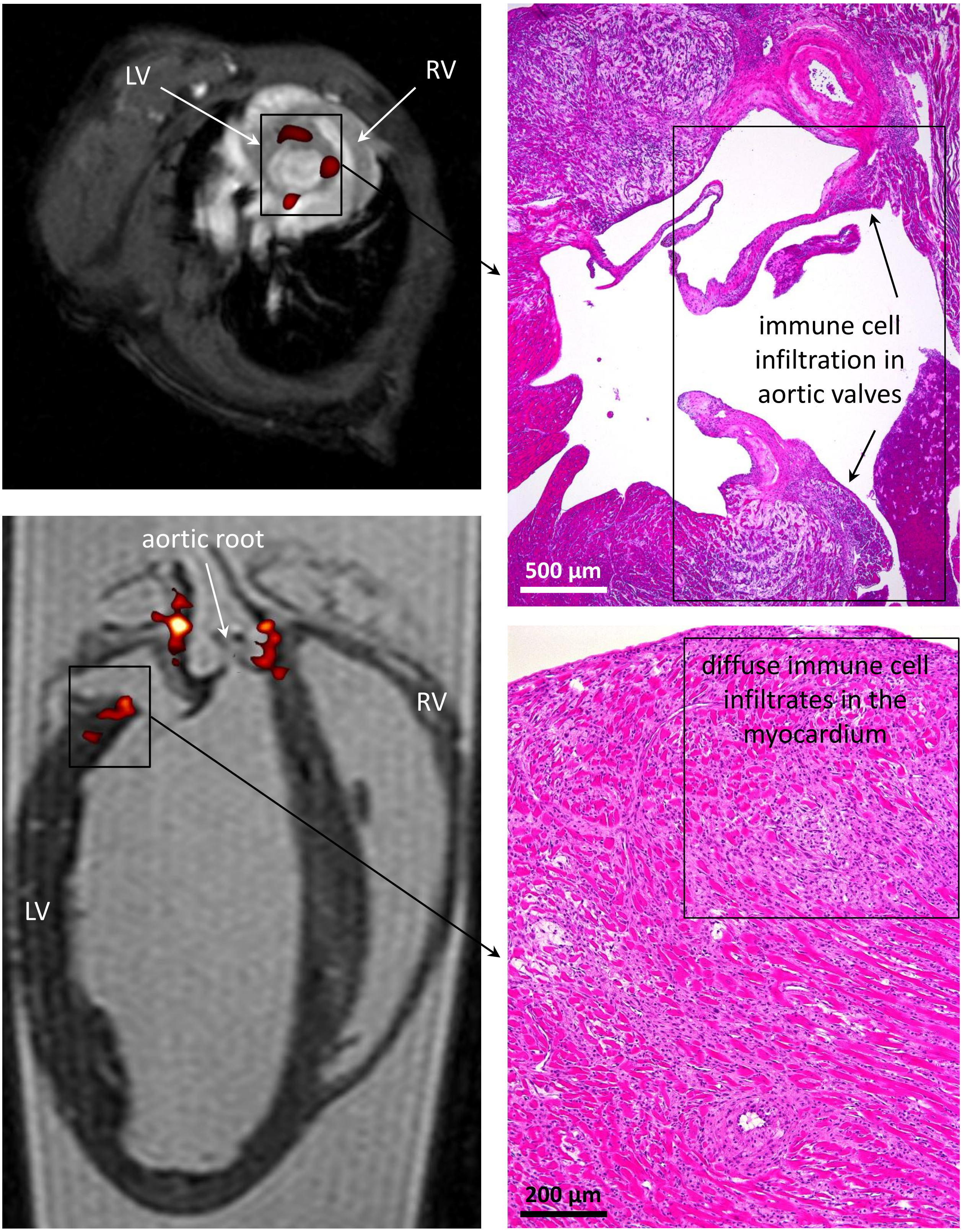

Supplementary figure 4: Validation of the ^{19}F signal and presence of immune cells in vicinity of the coronary arteries on day 10 after onset of the diet.

Left: In vivo short axis (top) and subsequent ex vivo long axis (bottom) ^{1}H/^{19}F MRI from the same animal sacrificed on day 10 after onset of the diet demonstrated that the ^{19}F label was primarily located at the base of the heart in close vicinity of the large coronary arteries indicating infiltration of PFC-loaded immune cells into inflammatory hotspots within the coronaries plaques (LV, left ventricle; RV right ventricle). **Right:** Confirmation of the presence of immune cells in anatomically corresponding histologic slices (top: valve level, 4-fold magnification; bottom: heart base, 10-fold magnification). Of note, at this early time point there are no histological signs for myocardial infarction.

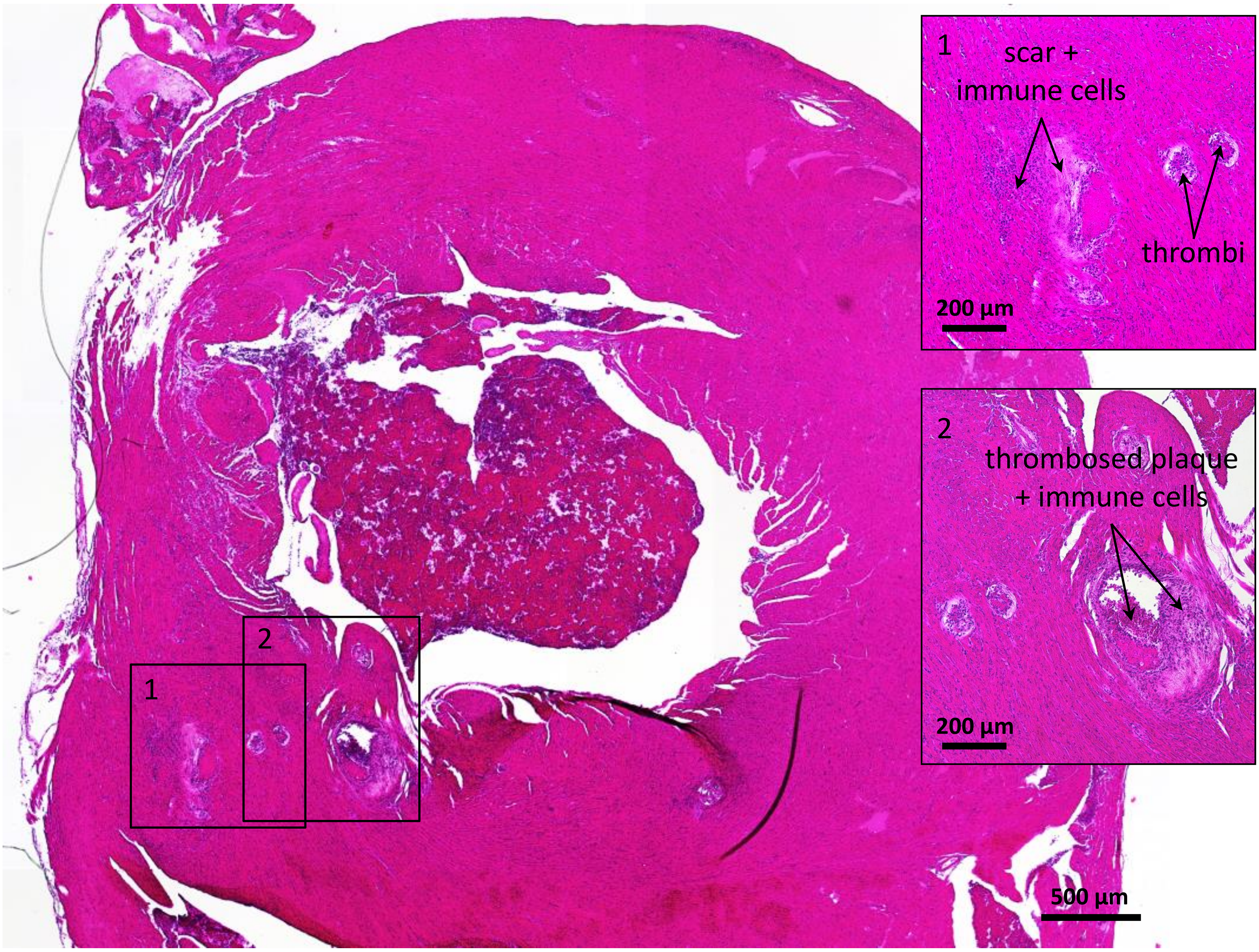

Supplementary figure 5: Histologic coregistration of scar and immune cells on day 15 after onset of the diet

Histological sections from the basal part of the heart with coregistration of infarcted myocardium (scar) and immune cells (insert 1) as well as thrombosed plaques and immune cells (insert 2). Overview is 4-fold, inserts are 10-fold magnifications.

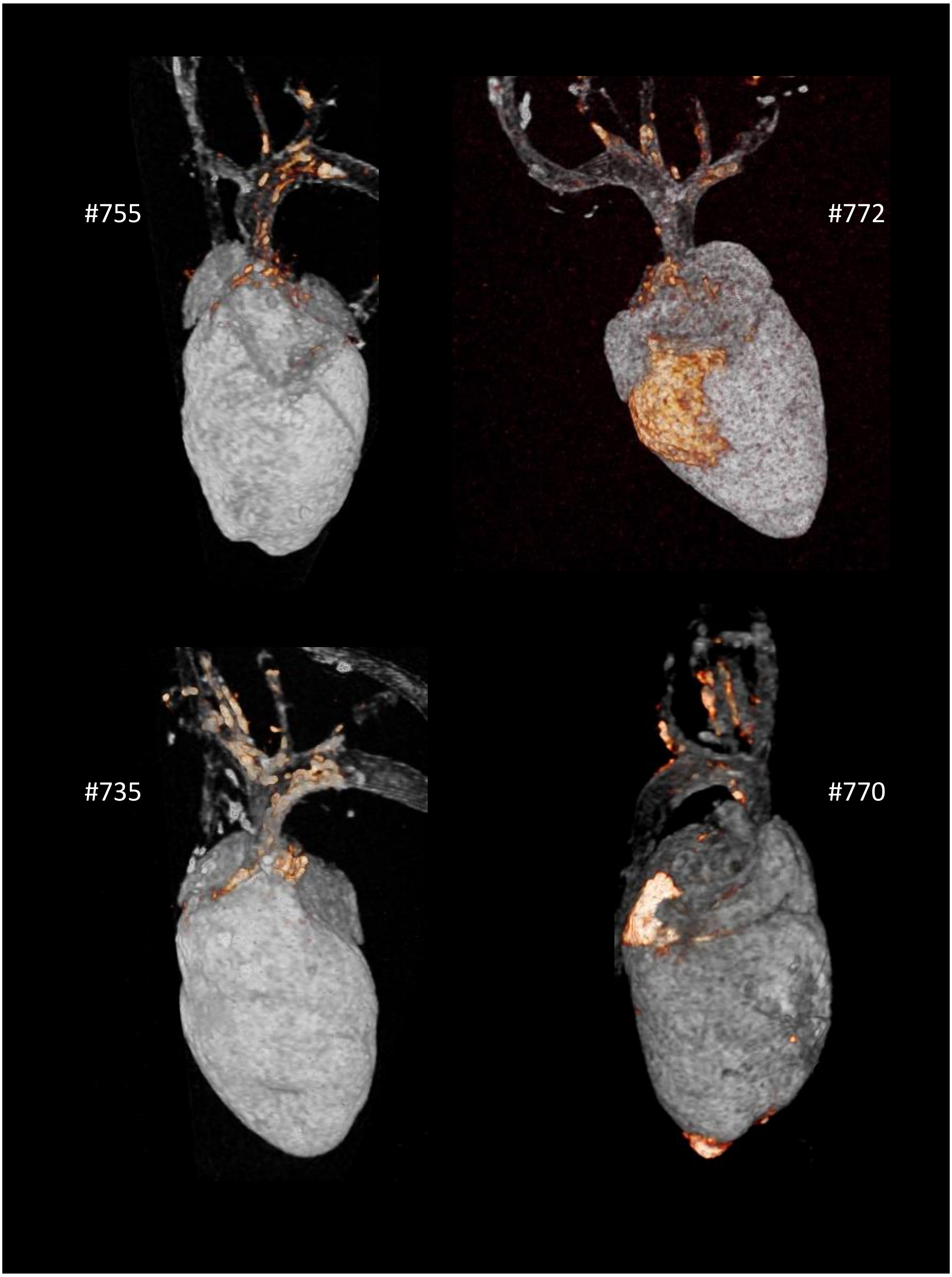

Supplementary figure 6: Variability of the phenotye after onset of the diet

Post mortem high resolution 3D $^{1}H/^{19}F$ MRI of four different animals sacrificed on day 15 after onset of the diet demonstrates the variability of the extent of inflammatory lesions at aortic branches and heart base as well as apex.

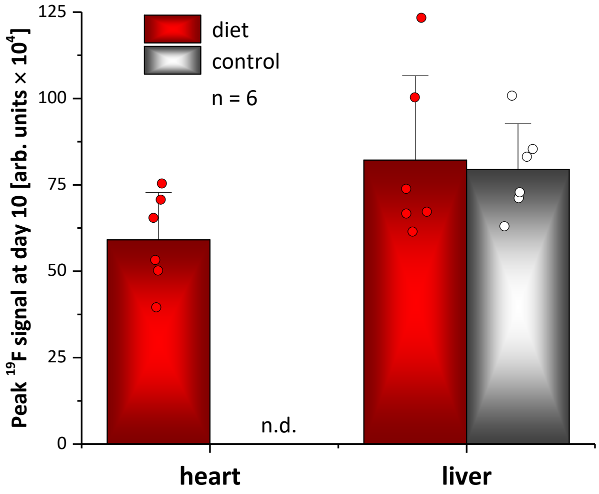

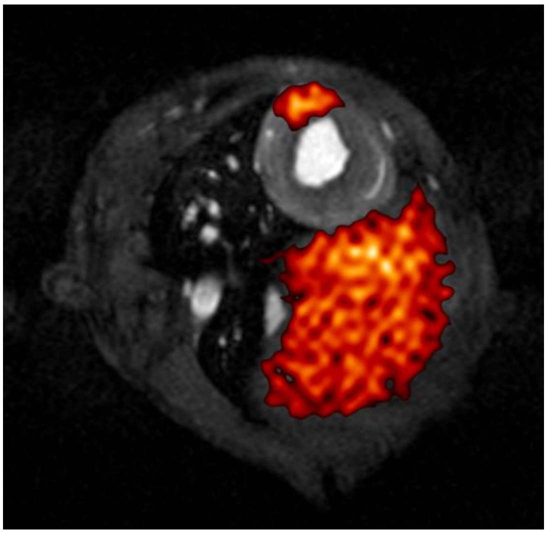

Supplementary figure 7: Lesion-specific cardiac ^{19}F signals in diet-exposed mice

Top: Quantification of the peak ^{19}F signal in inflamed myocardium *vs.* reticuloendothelial accumulation in the liver of mice exposed for 10 days to the diet or normal chow. Detectable ^{19}F signals in the heart were restricted to mice subjected to the atherogenic diet, while passive PFC deposition in the liver was equal in both groups. **Bottom:** Representative example of a mouse 10 days after onset of the diet illustrating a comparable ^{19}F signal intensity in inflamed myocardium as compared to the liver as major site of PFC deposition. In all other ^{19}F MR images, signals originating from the liver were masked for the sake of clarity.

Data are means ± SD of n = 6 independent experiments. Source data are provided as a source data file.

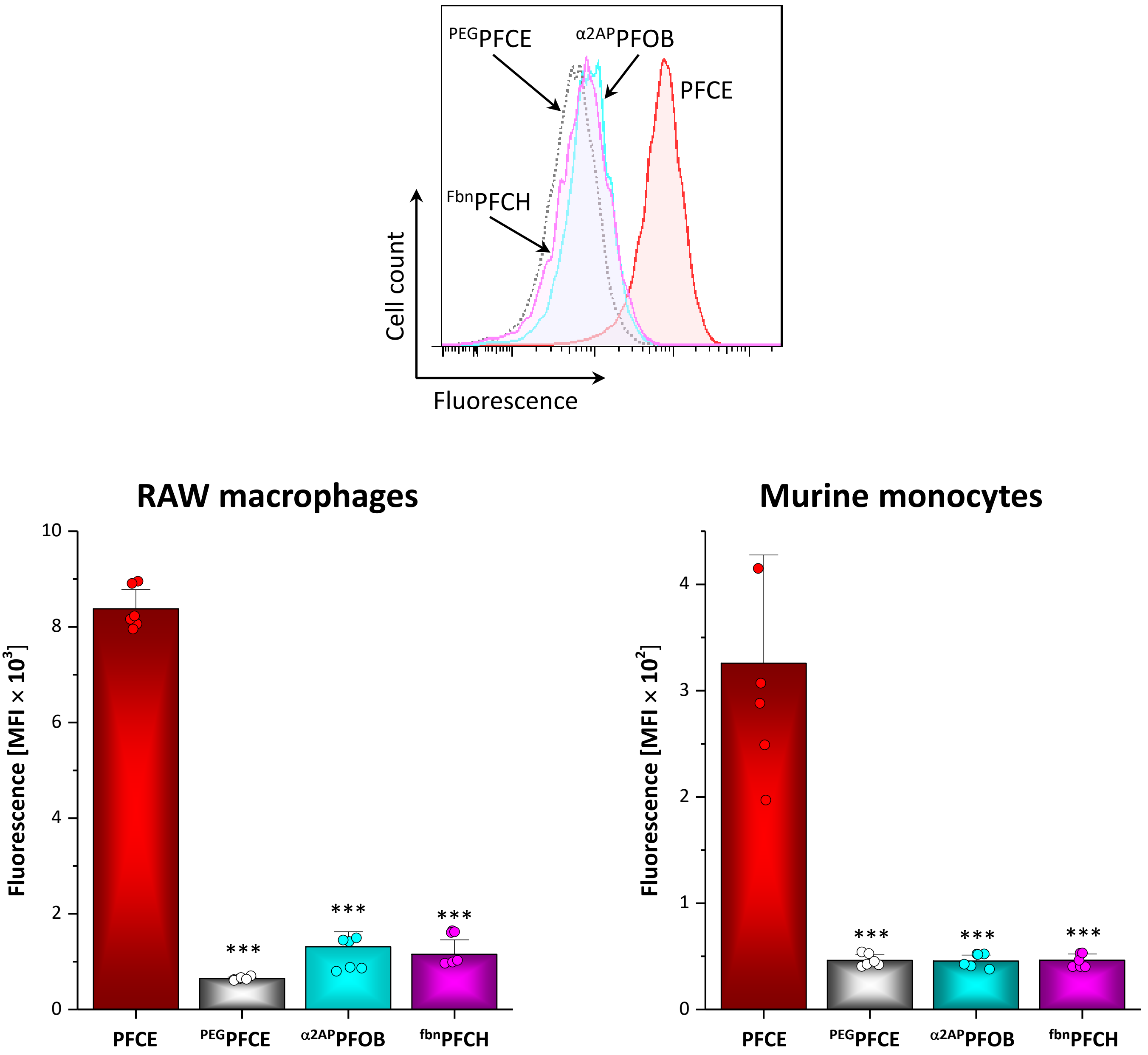

Supplementary figure 8: PEGylation blunts PFC uptake by phagocytic cell types

RAW macrophages or immune cells isolated from murine blood were incubated for 20 min with neat (PFCE, red) PEGylated (^{PEG}PFCE, grey) and PEGylated functionalized ($^{\alpha 2AP}$PFOB, cyan or ^{fbn}PFCH, magenta) Atto647-labelled PFCs and subsequently analysed by flow cytometry. For this, murine blood immune cells were stained for CD11b and Ly6G to identify monocytes. **Top:** The histogram plots show the Atto647 fluorescence signal of RAW macrophages incubated with PFCE (red), ^{PEG}PFCE (grey, dotted line) and PEGylated $^{\alpha 2AP}$PFOB (cyan) as well as ^{fbn}PFCH (magenta). **Bottom:** Quantification of PFC uptake by RAW macrophages (left) and murine monocytes (right) revealed the sucessful cloaking of PFCs by PEGylation resulting in a massive reduction (~85%) of PFC incorporation by phagocytic cell types.

Data are means ± SD of n = 6 independent experiments; *** = $P < 0.001$ with $P = 1.099 \times 10^{-12}$ (^{PEG}PFCE), 2.041×10^{-11} ($^{\alpha 2AP}$PFOB), and 2.899×10^{-11} (^{fbn}PFCH) for RAW macrophages and $P = 1.108 \times 10^{-4}$ (^{PEG}PFCE), 1.088×10^{-4} ($^{\alpha 2AP}$PFOB), and 1.111×10^{-4} (^{fbn}PFCH) for murine monocytes. Statistical significance was assessed by Student's two-sided t test compared to unPEGylated PFCE. Source data are provided as a source data file.

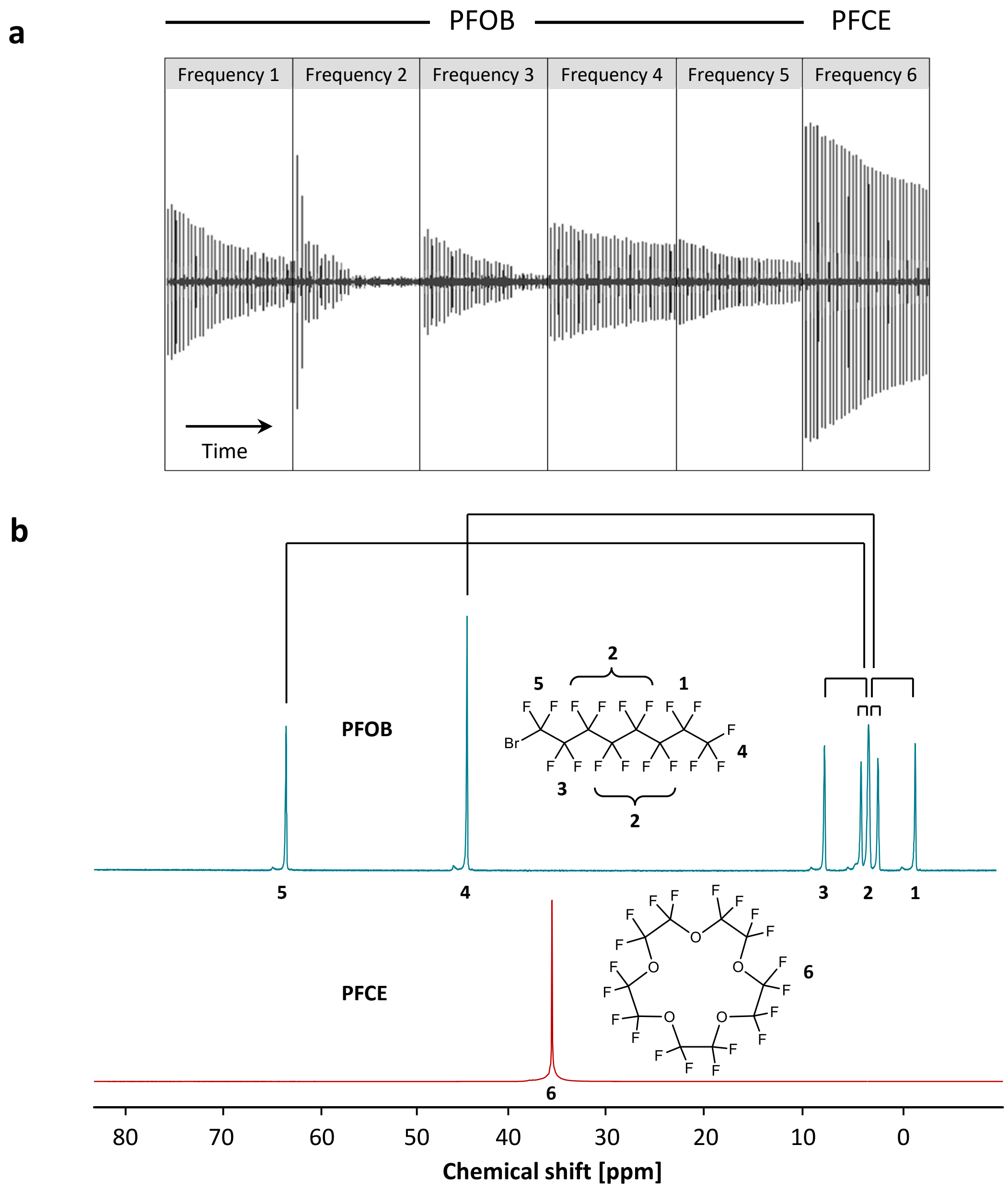

Supplementary figure 9: Minor effect of J modulation on signals used for multicolor ^{19}F MRI

a Acquisition windows for individual excitation frequencies of PFOB and PFCE. In the absence of any phase encoding gradient, the contours of the echo trains (each consisting of 32 echoes according to the RARE factor) represent the apparent T_2 decay of the corresponding resonances. Obviously, there is an accelerated decay for frequency 2 as compared to all other frequencies which is most likely caused by homonuclear J modulation effects which are well-known to occur in the linear part of the carbon skeleton of PFCs. As derived from 2D MR spectroscopy experiments[19] the strongest coupling occurs over four bonds ($^4J_{FF}$), and all possible coupling partners of this type are displayed in the PFOB spectrum in B). In fact, J modulation-induced signal loss can be effectively suppressed by chemical shift selective excitation and/or refocussing[39]. Nevertheless, simultaneous excitation of coupling partners at frequency 2 unavoidably leads to an accelerated signal decay as compared to the other frequencies. Hence, the relative signal intensity of the ^{19}F atoms excited in this region is lower than simply expected from their fraction of 47% in PFOB. However, this effect is negligible for frequencies 4 and 5 used in multicolor experiments. **b** ^{19}F MR spectra and signal assignments for PFOB and PFCE indicating the excitation frequencies 1-6 used in a. The diagram shows all combinations of $^4J_{FF}$ coupling in PFOB as a potential source of J modulation-induced signal loss.

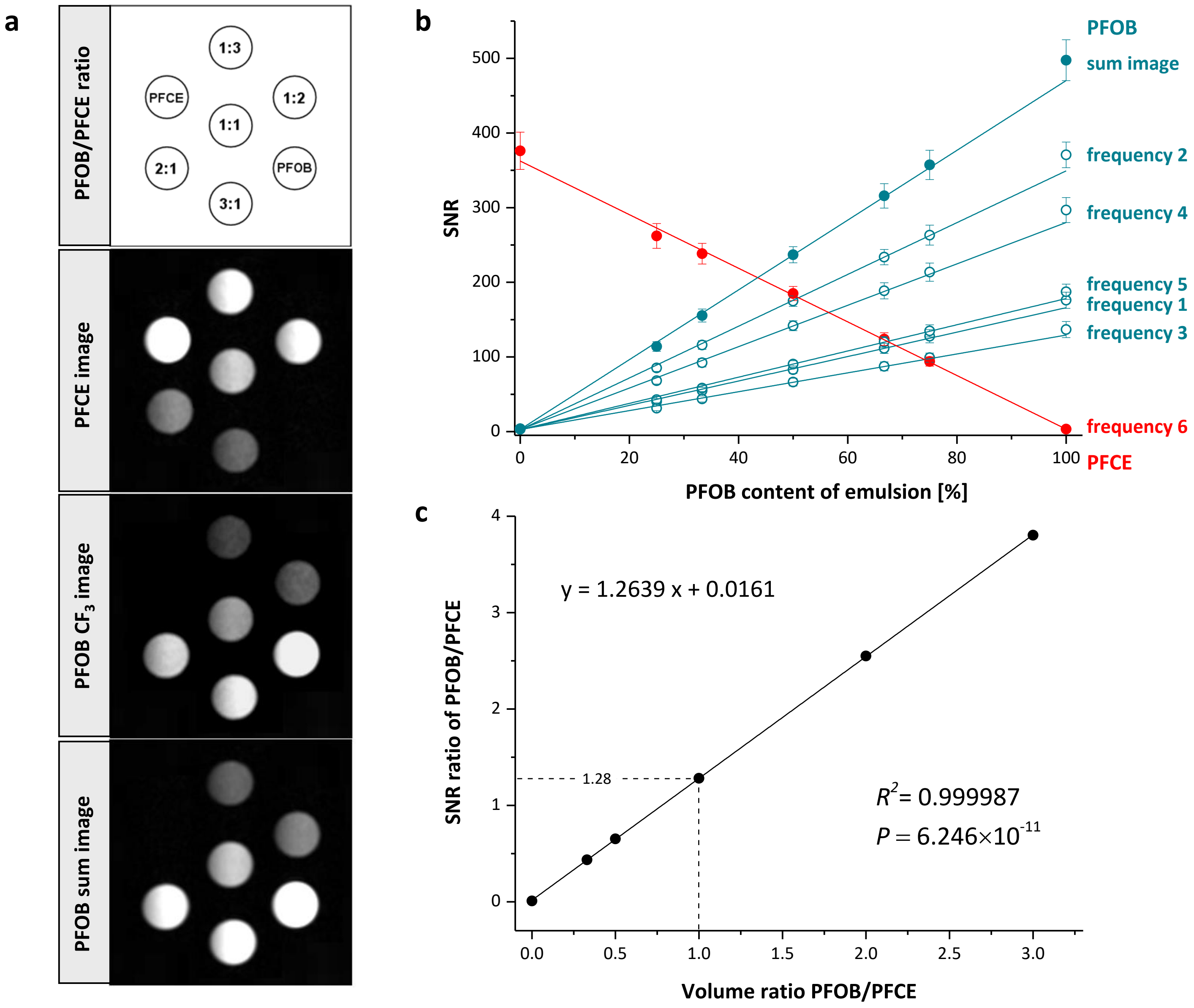

Supplementary figure 10: Reliable quantification by ^{19}F mCSSI

a Top: Scheme illustrating the orientation of tubes containing identical volumes but different ratios of PFOB and PFCE emulsions. Below: Simultaneously acquired ^{19}F mCSSI images of PFCE, of the CF_3 signal of PFOB, and after summation of all PFOB signals (scaled to identical noise levels). **b** Linear regressions for PFCE (red) and PFOB intensities (cyan; open circles: individual frequencies, closed circles: sum image) as a function of PFOB fraction (R^2 > 0.996 for all regressions). **c** Regression of SNR ratio against volume ratio (PFOB/PFCE).

In b error bars represent the standard deviation of the mean ^{19}F signal intensity over the respective region of interest (90 voxel). Source data are provided as a source data file.

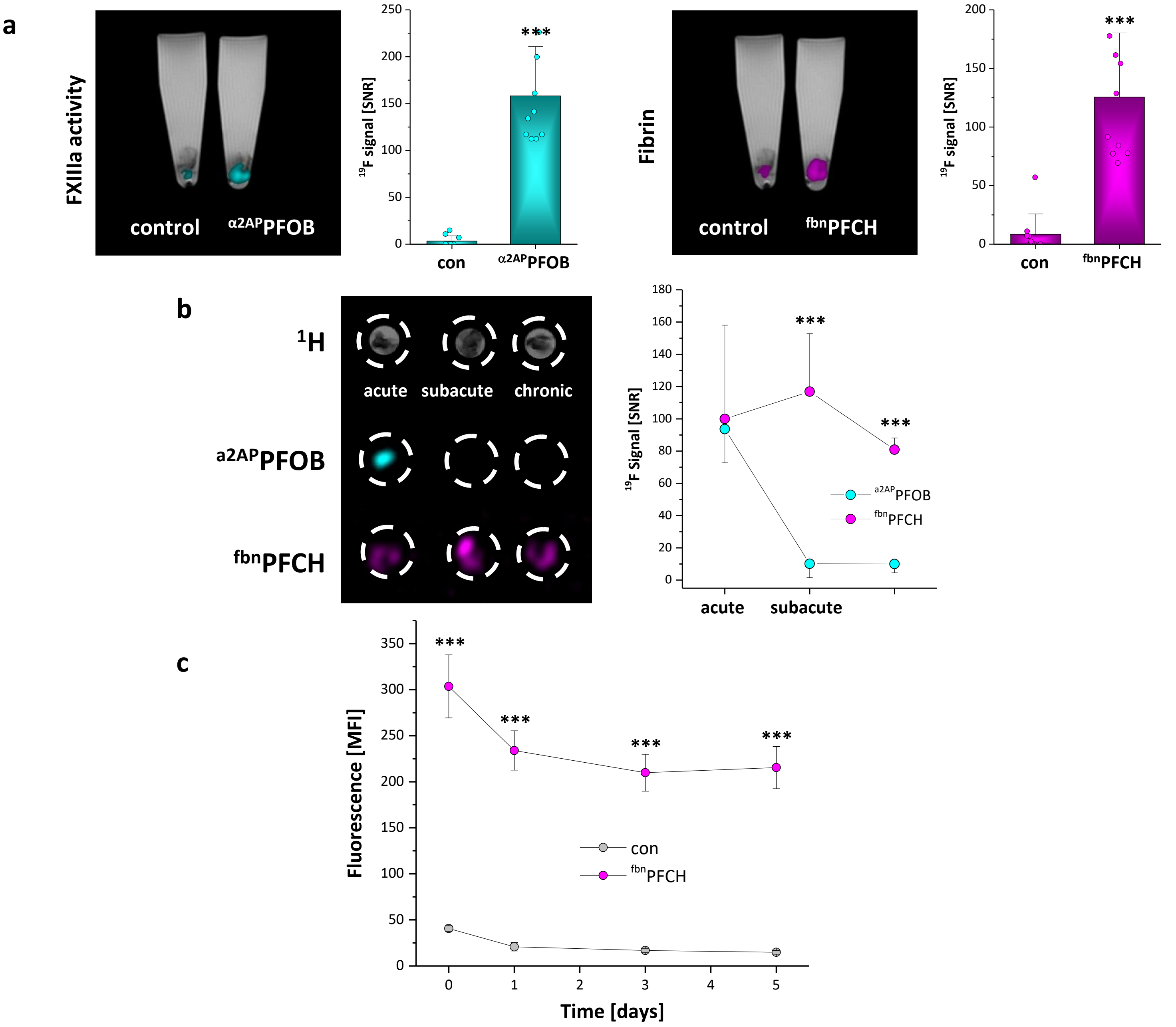

Supplementary figure 11: Binding of $^{\alpha 2AP}$PFOB and ^{fbn}PFCH to acute and chronic thrombi

Specificity of the thrombus-targeting approaches using PFCs modified with peptides directed against FXIIIa (α2AP) and fibrin (fbn). a ^{1}H/^{19}F MRI of freshly ex vivo generated human thrombi incubated with $^{\alpha 2AP}$PFOB or ^{fbn}PFCH. Images are longitudinal cross-sections through buffer-filled microfuges with the thrombi in the tip of the tube. Obviously, only thrombi exposed to the targeted PFCs display a clear ^{19}F signal and quantitative analysis revealed strongly enhanced binding of targeted as compared to control PFCs (con). **b** ^{19}F mCSSI of acute, subacute, and chronic thrombi incubated with both $^{\alpha 2AP}$PFOB and ^{fbn}PFCH. Images are axial cross-sections through three microfuge tubes and the ^{1}H image (top) shows the position of the thrombi. Note, that $^{\alpha 2AP}$PFOB binds to acute thrombi only, whereas subacute and chronic thrombi are also labelled with ^{fbn}PFCH. Subsequent quantification (right) confirmed these findings. **c** Binding of ^{fbn}PFCs to ex vivo generated thrombi over time, showing that ^{fbn}PFCs exhibit stable binding to aging thrombi.

Data are means ± SD of n = 10 (in a) or 5 (in b+c) independent experiments; *** = $P < 0.001$ with P = 2.956×10^{-8} ($^{\alpha 2AP}$PFOB) and 4.72×10^{-6} (chronic) vs. control in a, P = 4.112×10^{-4} (subacute) and 2.807×10^{-7} (chronic) for ^{fbn}PFCH vs. $^{\alpha 2AP}$PFOB in b and P = 3.272×10^{-7} (day 0), 4.954×10^{-8} (day 1), 5.910×10^{-8} (day 3), and 1.185×10^{-7} (day 5) for ^{fbn}PFCH vs. $^{\alpha 2AP}$PFOB. Statistical significance was assessed by Student's two-sided t test. Source data are provided as a source data file.

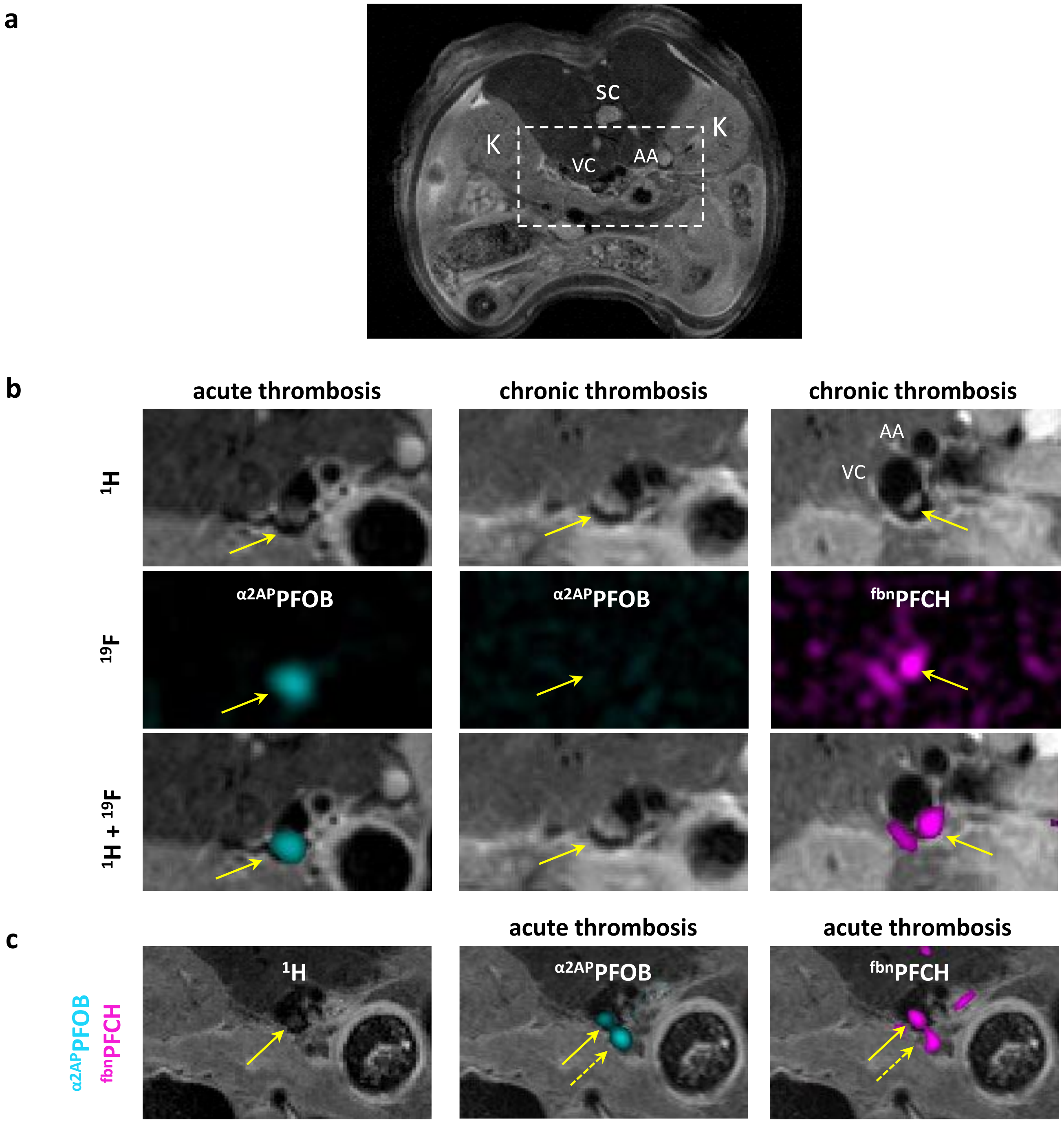

Supplementary figure 12: In vivo differentiation of acute and chronic thrombi by ^{19}F mCSSI in the vena cava

Artefact-free in vivo imaging of deep venous thrombi in the vena cava inferior ($FeCl_3$-induced) by ^{19}F mCSSI after injection of $^{\alpha 2AP}$PFOB and/or ^{fbn}PFCH. **a** Dashed lines in the ^{1}H image indicate the magnified areas surrounding the vena cava shown beneath (K = kidney, SC = spinal cord; VC = vena cava, AA = abdominal aorta). **b+c** Zoomed sections comprising VC and AA; yellow arrows indicate the location of the thrombus in the VC. **b** 1st column: Injection of $^{\alpha 2AP}$PFOB 5 min before induction of deep venous thrombosis visualizes the acutely formed thrombus. 2nd column: No detectable signal for $^{\alpha 2AP}$PFOB when injected 24 hrs after thrombus induction. 3rd column: Injection of ^{fbn}PFCH 24 hrs after induction of deep venous thrombosis unequivocally detects the thrombus in its advanced stage. **c** Co-administration of $^{\alpha 2AP}$PFOB and ^{fbn}PFCH immediately after induction of deep venous thrombosis resulted in congruent patterns for both PFCs as detected by ^{19}F mCSSI. Note, that in the anatomical ^{1}H image only a dark-grey structure in the VC indicates the presence of a thrombus, while ^{19}F MRI also clearly visualizes the transluminal part of the thrombus (dashed arrows).

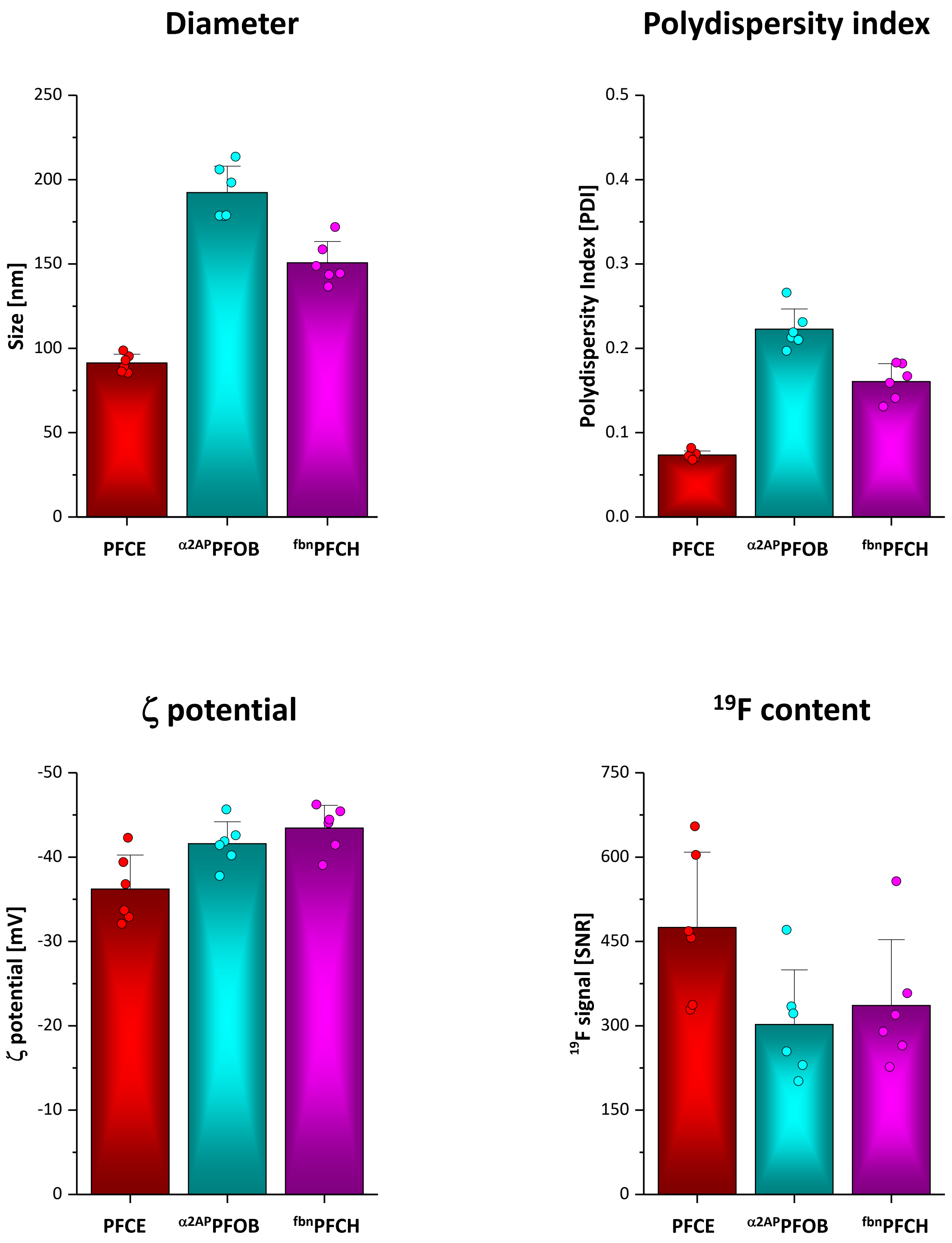

Supplementary figure 13: Physicochemical properties of the different PFCs used in this study

PFCs were analyzed by dynamic light scattering (DLS) to determine the hydrodynamic diameter, the polydispersity index (PDI), and the ζ potential. The ^{19}F content was analyzed by ^{19}F mCSSI.

Data are means ± SD of n = 6 independent experiments. Source data are provided as a source data file.

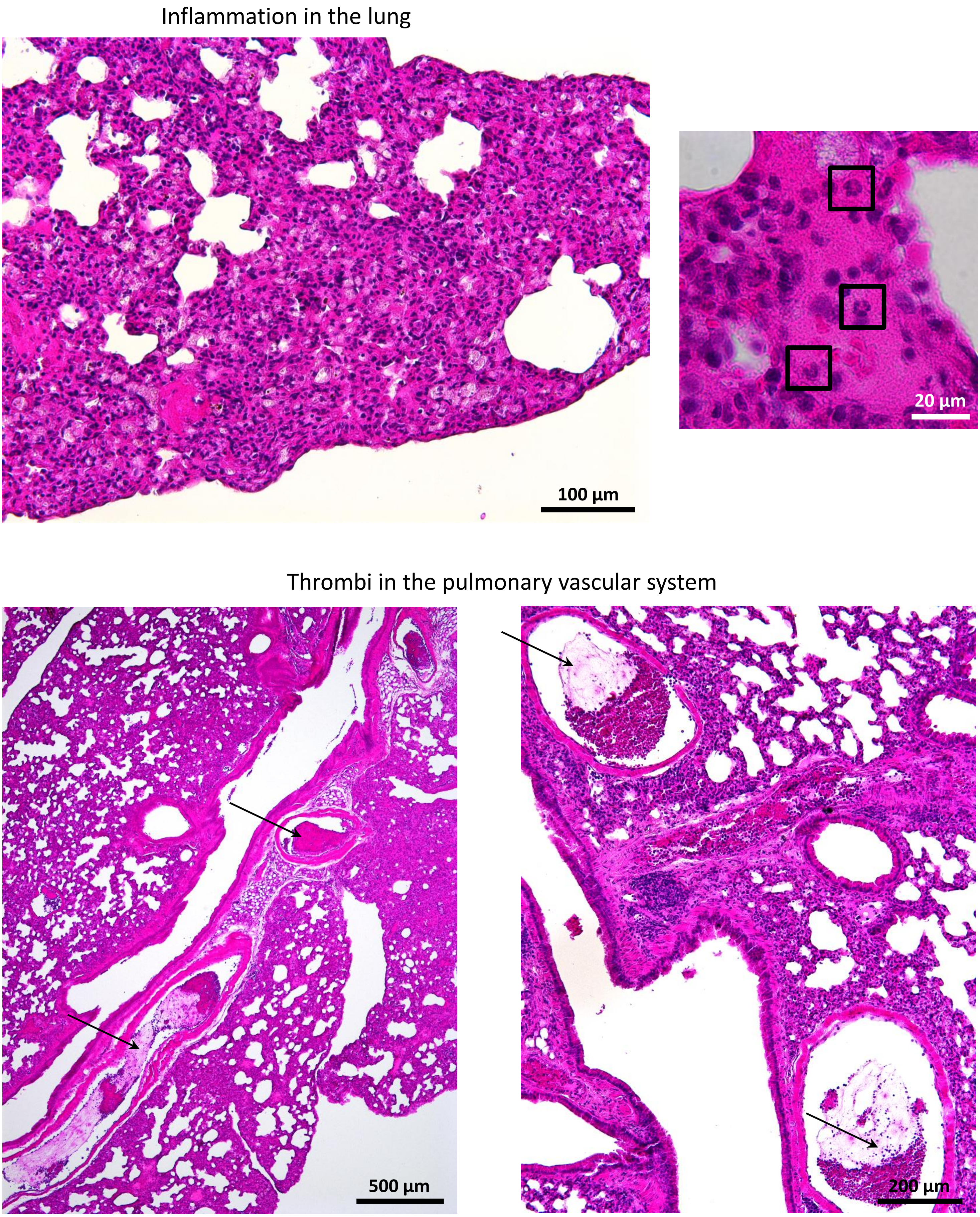

Supplementary figure 14: Histologic validation of both inflammation and thrombus development in the pulmonary system on day 10 after onset of the diet

Top: Detection of leukocytes (rectangles) in the peripheral lung (left 40-fold, right 100-fold magnification). The zoomed section is located outside the overview on the left. **Bottom:** Formation of thrombi (arrows) in pulmonary vessels (left 4-fold, right 10-fold magnification).

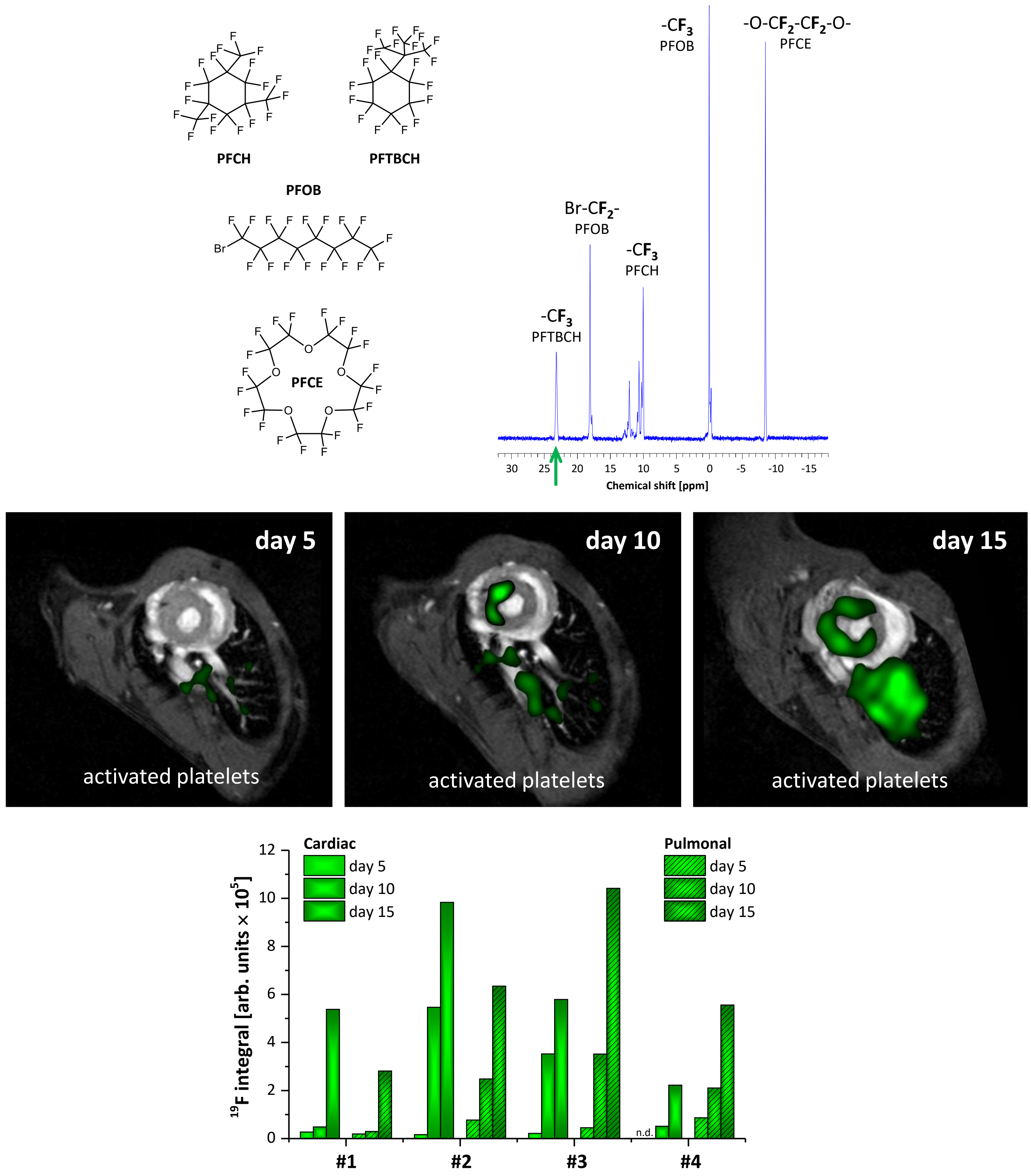

Supplementary figure 15: Extending the approach for more colors and activated platelets

Top: Structures and ^{19}F MR spectrum of a PFC mix containing PFCE, PFOB, PFCH and perfluoro(tert-butylcyclohexane) (PFTBCH) showing clearly separated resonances for the individual PFCs. **Middle:** Equipping the latter with a unique human single-chain antibody (scFv[27], green), that specifically binds to the activated conformation of GPIIb/IIIa enabled us to track also the progressive accumulation of activated platelets in heart and lung of mice exposed to the diet. **Bottom:** Temporal development of ^{19}F signals in the heart and pulmonal system of four diet-fed animals (#1 – #4) receiving repetitive injections (see supplemental Figure S1) of scFv-targeted PFCs demonstrating again the largely varying phenotype of the model with individually heterogenous ^{19}F patterns in heart or lung. Source data are provided as a source data file.

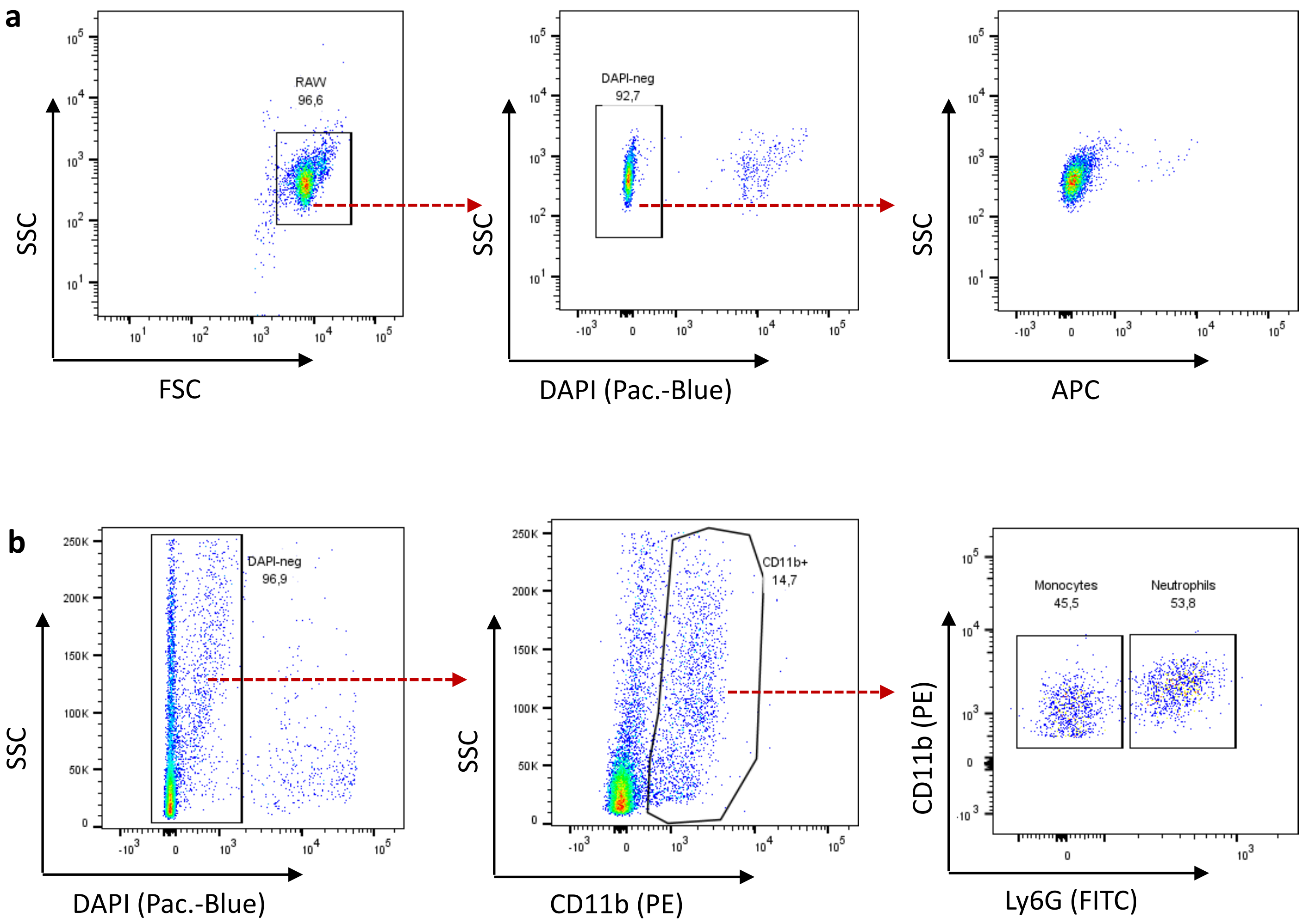

Supplementary figure 16: Gating scheme for RAW macrophages and murine monocytes

Representative pseudocolor dot plots that display the gating of RAW macrophages in a and murine monocytes in b**. a** Left: FSC/SSC profile of RAW macrophages; middle: DAPI staining to exclude dead cells; right: SSC/APC plot to display the cells in absence of PFC labelling. **b** Left: SSC/DAPI staining to exclude dead cells; middle: SSC/CD11b-PE plot and CD11b-PE/Ly6G-FITC dot plot to discriminate neutrophils (CD11b$^+$/Ly6G$^+$) and monocytes (CD11b$^+$/Ly6G$^-$) as shown on the right.

# Frequency	PFC	Assignment	Absolute frequency [MHz]	Chemical shift [ppm]
1	PFTBCH	-C**F**$_3$	376.5461748	23.15
2	PFOB	-CBr**F**$_2$	376.5442538	18.05
3	PFCH	-C**F**$_3$	376.5412238	10.00
4	PFOB	-C**F**$_3$	376.5374588	0
5	PFCE	-C**F2**-C**F**$_2$-	376.5342317	–8.57
6	PFCH	-C**F**-CF$_2$	376.4996848	–100.321

Supplementary table 1: Characteristics of excitation frequencies used for ^{19}F mCSSI

Chemical shifts were determined by non-selective spectroscopy and are given relative to the resonance frequency of the PFOB CF_3 group.
