# Supplementary material for: Multi-targeted 1H/19F MRI unmasks specific danger patterns for emerging cardiovascular disorders
Source: Nat Commun. 2021 Oct 6;12:5847. doi: 10.1038/s41467-021-26146-6 (PMC8494909; doi:10.1038/s41467-021-26146-6)
Supplement: Supplementary file 3 — Reporting Summary [file 41467_2021_26146_MOESM3_ESM.pdf]

nature research

Corresponding author(s): Ulrich Flögel

Last updated by author(s): 17.08.2021

Reporting Summary

Nature Research wishes to improve the reproducibility of the work that we publish. This form provides structure for consistency and transparency in reporting. For further information on Nature Research policies, see our Editorial Policies and the Editorial Policy Checklist.

Statistics

For all statistical analyses, confirm that the following items are present in the figure legend, table legend, main text, or Methods section.

n/a	Confirmed	
☐	☒	The exact sample size (n) for each experimental group/condition, given as a discrete number and unit of measurement
☐	☒	A statement on whether measurements were taken from distinct samples or whether the same sample was measured repeatedly
☐	☒	The statistical test(s) used AND whether they are one- or two-sided<br>*Only common tests should be described solely by name; describe more complex techniques in the Methods section.*
☒	☐	A description of all covariates tested
☐	☒	A description of any assumptions or corrections, such as tests of normality and adjustment for multiple comparisons
☐	☒	A full description of the statistical parameters including central tendency (e.g. means) or other basic estimates (e.g. regression coefficient) AND variation (e.g. standard deviation) or associated estimates of uncertainty (e.g. confidence intervals)
☐	☒	For null hypothesis testing, the test statistic (e.g. F, t, r) with confidence intervals, effect sizes, degrees of freedom and P value noted<br>*Give P values as exact values whenever suitable.*
☒	☐	For Bayesian analysis, information on the choice of priors and Markov chain Monte Carlo settings
☒	☐	For hierarchical and complex designs, identification of the appropriate level for tests and full reporting of outcomes
☐	☒	Estimates of effect sizes (e.g. Cohen's d, Pearson's r), indicating how they were calculated

Our web collection on statistics for biologists contains articles on many of the points above.

Software and code

Policy information about availability of computer code

Data collection	ParaVision 5.1
Data analysis	ParaVision 5.1, LabVIEW 6, OriginPro 2016, Amira 4.0, FlowJo, Microtrac Flex Software

For manuscripts utilizing custom algorithms or software that are central to the research but not yet described in published literature, software must be made available to editors and reviewers. We strongly encourage code deposition in a community repository (e.g. GitHub). See the Nature Research guidelines for submitting code & software for further information.

Data

Policy information about availability of data

All manuscripts must include a data availability statement. This statement should provide the following information, where applicable:

- Accession codes, unique identifiers, or web links for publicly available datasets
- A list of figures that have associated raw data
- A description of any restrictions on data availability

All data necessary to understand and assess the conclusions of the manuscript are present in the paper or the supplementary materials. Raw MRI data are available on request.

Field-specific reporting

Life sciences study design

All studies must disclose on these points even when the disclosure is negative.

Sample size	No statistical methods were used to predetermine sample size. Data were analyzed in a continous manner with a minimum of n=6 per group. Statistical significances found for the experiments carried out were usually in the range of P < 10^-5, thus no further replicates were required.
Data exclusions	No data were excluded.
Replication	All attempts at replication were successful with statistical significances in the range of P < 10^-5.
Randomization	The experiments were not randomized. The general phenotype of the used mouse strain is well known and already documented in Refs. 16 + 17. Furthermore, no different treatment groups were involved. In the present study, we just aimed to characterize the disease progression by non-invasive MRI techniques. Thus, we felt that no randomization or blinding of animals was required.
Blinding	The investigators were not blinded during experiments and outcome assessment. he general phenotype of the used mouse strain is well known and already documented in Refs. 16 + 17. Furthermore, no different treatment groups were involved. In the present study, we just aimed to characterize the disease progression by non-invasive MRI techniques. Thus, we felt that no randomization or blinding of animals was required.

Reporting for specific materials, systems and methods

We require information from authors about some types of materials, experimental systems and methods used in many studies. Here, indicate whether each material, system or method listed is relevant to your study. If you are not sure if a list item applies to your research, read the appropriate section before selecting a response.

Materials & experimental systems

n/a	Involved in the study	
☐	☒	Antibodies
☒	☐	Eukaryotic cell lines
☒	☐	Palaeontology and archaeology
☐	☒	Animals and other organisms
☐	☒	Human research participants
☒	☐	Clinical data
☒	☐	Dual use research of concern

Methods

n/a	Involved in the study	
☒	☐	ChIP-seq
☐	☒	Flow cytometry
☒	☐	MRI-based neuroimaging

Antibodies

Antibodies used	CD11b (CD11b-PE, clone M1/17; BD Biosciences) and Ly6G (Ly6G-FITC, clone 1A8, BioLegend).
Validation	These mAbs are rountinely used in our lab for long times. Validation includes: Single staining and titration of the mAbs to determine optimal staining conditions.

Animals and other organisms

Policy information about studies involving animals; ARRIVE guidelines recommended for reporting animal research

Laboratory animals	Mice expressing a hypomorphic mutant form of ApoE (ApoE-Rh/h) and lacking the scavenger receptor class B type I (SR-BI-/-) were bred and housed with a 12L/12D cycle at an ambient temperature of approximately 22 °C and a humidity of 55% at the central animal facility of the Heinrich Heine University Düsseldorf, Germany. Mice were fed a standard chow diet and received tap water ad libidum. Male and female mice with 25-30 g body weight and 8-10 weeks of age were used.
Wild animals	This study did not involve wild animals.
Field-collected samples	The study did not involve samples collected from the field.
Ethics oversight	Animal experiments were performed in accordance with the European Union guidelines described in the directive 2010/63/EU and were approved by North Rhine Westphalian State Agency for Nature, Environment and Consumer Protection (LANUV = Landesamt für Natur, Umwelt und Verbraucherschutz Nordrhein-Westfalen), Germany.

Note that full information on the approval of the study protocol must also be provided in the manuscript.

Human research participants

Policy information about studies involving human research participants

Population characteristics	Healthy human volunteers; age: 18-49; informed consent; criteria for exclusion: Platelet- or coagulation-dysfunctions; therapy with platelet inhibitors (ASS, clopidogrel); acute/chronic infectious diseases or other major acute inflammatory diseases; pregancy.
Recruitment	From the campus or from neighboring departments/groups.
Ethics oversight	Ethics Comittee of the Heinrich Heine University, Düsseldorf, Germany.

Note that full information on the approval of the study protocol must also be provided in the manuscript.

Flow Cytometry

Plots

Confirm that:

- [x] The axis labels state the marker and fluorochrome used (e.g. CD4-FITC).
- [x] The axis scales are clearly visible. Include numbers along axes only for bottom left plot of group (a 'group' is an analysis of identical markers).
- [x] All plots are contour plots with outliers or pseudocolor plots.
- [x] A numerical value for number of cells or percentage (with statistics) is provided.

Methodology

Sample preparation	RAW cells: cultivated macrophage cell line; Murine monocytes: Blood from inferior vein, erythrocytes lysis
Instrument	FACS Canto II
Software	FACS Diva ; FlowJo
Cell population abundance	RAW cells: 90-95% viable cells ; Murine monocytes: 40-50% of CD11b+ blood leukocytes
Gating strategy	RAW: DAPI positive cells excluded; Murine monocytes. FSC/SSC profile ; DAPI negative ; CD11b+ and Ly6G-negative

- [x] Tick this box to confirm that a figure exemplifying the gating strategy is provided in the Supplementary Information.
